# NMR spectroscopy in drug discovery and development: Evaluation of physico-chemical properties

**DOI:** 10.5599/admet.737

**Published:** 2019-12-11

**Authors:** Mire Zloh

**Affiliations:** UCL School of Pharmacy, University College London, 29/39 Brunswick Square, London, WC1N 1AX, UK; Faculty of Pharmacy, University Business Academy, Trg mladenaca 5, 21000, Novi Sad, Serbia

**Keywords:** logP, qNMR, drug solubility, DOSY, pharmaceutical formulations

## Abstract

Determination of physico-chemical properties of compounds is one of the corner-stones in selection of hit molecules for further progression into lead development in the modern drug discovery process. The speed of traditional analyses and limited quantities of hit molecules are obstacles for efficient acquisition of experimental data. Herein, the range of applications of quantitative nuclear magnetic resonance (NMR) spectroscopy as a fast technique used to evaluate solubility, log P and pKa are discussed.

## Introduction

The process of modern drug discovery and development is highly complex process that aims to create new medicines that meet stringent requirements set by governmental bodies, such as Food and Drug Administration (FDA) in the USA, European Medicines Agency (EMA) and others around the world [1]. Efforts of the industry have already heavily utilized most of the easiest disease targets, which reduces the chances of serendipity in discovery of new therapeutic agents. This requires systematic approaches in identifying new targets or paradigm shift in finding novel lead molecules, resulting in the need for iterative, tedious and quite often expensive multidisciplinary processes. Although the record breaking number of 59 drugs, including 17 biologics, were approved by FDA in 2018 [2], the high failure rates of clinical candidates frustrates pharmaceutical industry and academic research groups. It is shown that safety issues are linked to off-target interactions of these compounds [3] or their physico-chemical properties [4].

Therefore, pharmaceutical industry was focused on the drug likeness properties of drug candidates in recent years. Absorption, distribution, metabolism, excretion, and toxicity (ADMET) properties are generally included as filters during process of identification of hit molecules or a guide in the design or modifications of lead molecules. A significant progress was achieved in prediction of ADMET properties using *in silico* methods and these approaches are generally cheaper and greener when compared to their experimental determination [5]. Although these methods can be used in virtual high throughput screening as filtering steps, they are often limited by their applicability domains, as prediction of ADMET molecules with novel scaffolds outside of chemical space of training sets may not be reliable. Most relevant assessments of compounds properties can be achieved by employing *in vivo* assays, often preceded by *in vitro* experiments as indicators of their ADME fate [6]. Such two-tier approaches cannot always be employed for large number of compounds or in a high throughput manner, which demands utilization of biophysical techniques and development of novel assays to evaluate physico-chemical characteristics in relation to ADMET properties. Significant progress has been achieved in utilization of chromatography to measure lipophilicity of compounds and their biomimetic properties [7,8], as well as to evaluate their polarity, conformational states and intramolecular hydrogen bonds [9]. The development of great variety of chromatographic methods is the result of possibility to utilize a range of stationary phases and to vary mobile phases to represent different parts of biological systems [8], while the automation increases the throughput of analysis.

On the contrary, nuclear magnetic spectroscopy (NMR) spectroscopy is not readily associated with measuring ADMET properties. Up to date, the Scopus search using keyword “NMR” in conjunction with “ADME” results in 125 records, which is significantly lower when compared to 456 when the “chromatography” keyword is used. This may be a consequence that NMR requires expensive instrumentation and samples are generally dissolved in deuterated solvents at concentrations that may be considerably higher than those observed in physiological conditions. Therefore, this spectroscopic technique is mainly utilized to acquire qualitative information on molecular structure of organic compounds as well as biopolymers, or for the assessment of the sample purity.

However, NMR can also provide information on three dimensional (3D) structures at atomic level resolution of pure samples and mixtures, with a possibility to detect and even quantify intermolecular interactions as dynamic processes, making this techniques versatile and applicable to different stages in the search for new therapeutic agents [10]. Therefore, despite its disadvantages, NMR spectroscopy plays a significant role in the drug discovery and development processes. Some of these applications include: protein structure determination in solution [11] or target structure validation in presence of membranes [12], enabling structure-based drug discovery in solution [13,14] or In-Cell [15], identification of hit compounds [16] and determination of their conformations [17], development of biopharmaceutical formulations [18], and evaluation of drug metabolism and toxicity in patients [19,20] that can even play part in post-market surveillance [21,22].

It appears that the above applications may not be generally implemented as an integral part of research programs in pharmaceutical industry [10]. Therefore, the information that can be readily obtained by NMR should be further integrated in the drug discovery process and wider utilized for the evaluation of the physico-chemical properties. Herein, simpler applications of hydrogen nuclear magnetic spectroscopy (^1^H NMR) spectroscopy, not often reported as a tool for evaluating some ADMET properties, are explored.

## NMR

### Quantitative NMR (qNMR)

Most of the ADME properties rely on concentration measurements, where quantitative NMR (qNMR) can play a significant role in the pharmaceutical industry. One of the major advantages of the NMR spectroscopy is its inherent quantitative nature of the signal, as the measured signals can be directly proportional to the number of investigated nuclei in the same environment. These signals can be obtained for nuclei most relevant to pharmaceutical industry, such as ^1^H, ^13^C, ^19^F and ^31^P. Although the acquisition of ^19^F and ^31^P NMR signals results in high specificity analysis, the ^1^H signal is most commonly used in qNMR spectroscopy due to its presence in most drugs, short relaxation times and higher sensitivity when compared to other nuclei. The adequate setup of the experiment (pulse sequence that allows nuclei to fully relax and to minimize nuclear Overhauser effect) will result in an one dimensional (1D) NMR spectrum with areas under peaks proportional to concentrations of sample components. This phenomenon is observed for all molecules that contain investigated nuclei, as the NMR does not depend on compound-specific properties, such as presence of a chromophore, chemical structure or volatility. Furthermore, if a sample is fully dissolved, concentrations of all compounds with NMR active nuclei can be determined using a single standard, without reliance on specific reference standards for each compound [23]. The qNMR can be used as a relative quantification method by evaluating molar ratio between two compounds (analyte and referent compound) in a solution (Eq. 1), or it can be used to determine absolute content of the analyte of a mixture if the concentration of the referent compound is known (Eq. 2):


(1)

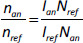




(2)

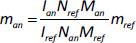



where *n* is the number of mols, *I* is the area under the peak that correspond to a signal, *N* number of nuclei contributing to the signal, *M* is molar mass and *m* is the gravimetric mass. The above approach does not require calibration curves or isolation of compounds.

**Figure 1.**
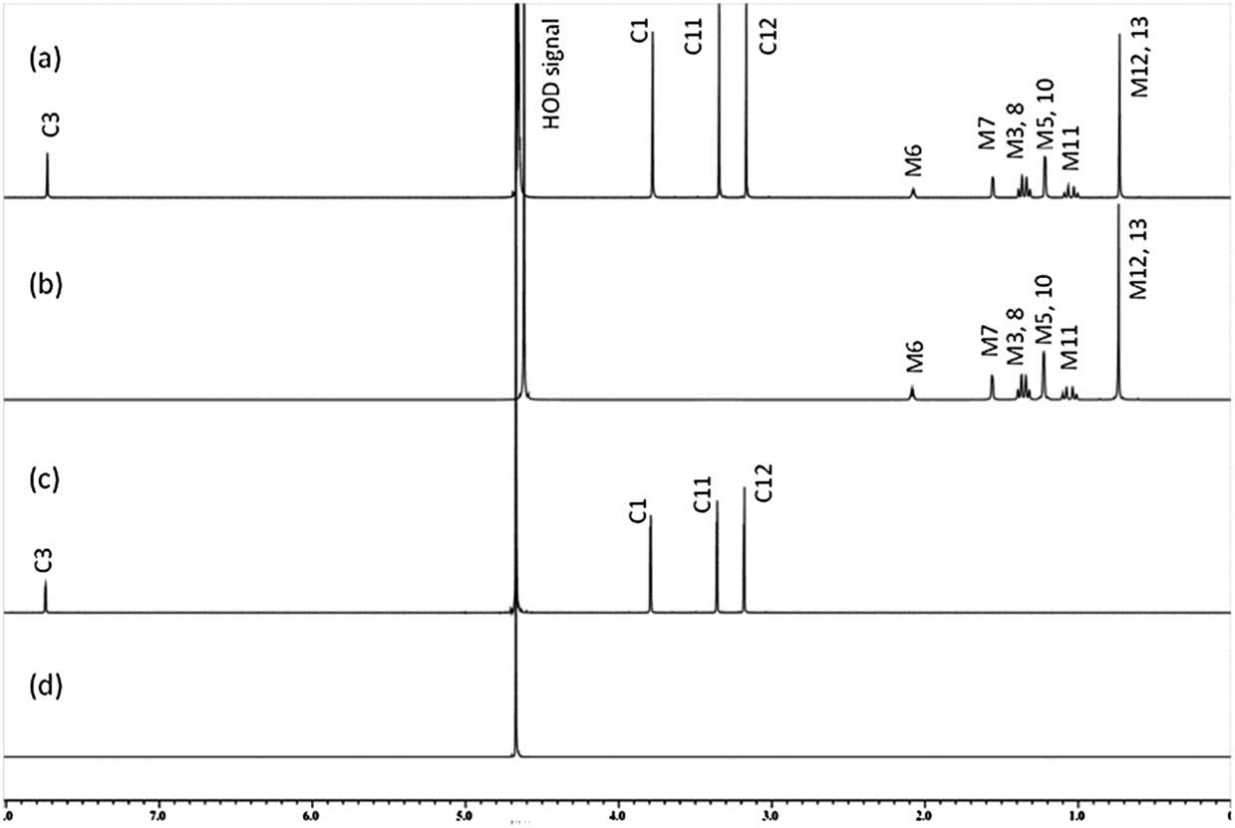
Overlay of ^1^H NMR spectra of memantine hydrochloride from tablets in solution **(a)**, drug standard **(b)**, internal standard **(c)**, and blank **(d)**. All spectra were acquired with same parameters and processed similarly. Taken with permission from [25]. Copyright (2016) with permission from Wiley.

Multicomponent mixtures can be subjected to direct and simultaneous determination of concentrations of several molecules as long as it is possible to find a characteristic signal for each analyte. For example, in a case of quantification of caffeine, formic acid, trigonelline and 5-HMF in soluble coffees, singlet peaks for molecule were identified that were not overlapping with the signals from the other components present in the sample. That allowed quick and reliable measurement of their concentrations with the limits of the detection for each component that were between 0.3 mg to 1.3 mg per gram of solid product [24]. In pharmaceutical industry setting, impurities (including toxic compounds) [25] or components of formulations can be quantified at the same time with measurement of the concentration of the active pharmaceutical ingredient. The quantification of memantine hydrochloride from tablets dissolved in D_2_O shown a good separation between a selected signal from signal (three hydrogens giving a singlet at 0.75 ppm) and caffeine signal used as a referent standard (three hydrogens giving a singlet at 3.13 ppm) (Figure 1). The method was validated according to ICH guideline Q2 (R1) with demonstrated recovery of 99.26 % and RSD of 0.38 % [25].

The advantages resulted in the of use of qNMR not only in analyses of samples of simple mixtures of small organic molecules, but also in the evaluation of complex mixtures found in the field of metabolomics and studies of physiological pathways. Presence of many compounds in such mixtures results in a difficulty to identify an isolated signal of the referent compound to ensure reliable quantification. To facilitate the optimal choice of referent compounds for a range of samples, a set of 15 molecules were tested for their properties and suitability for the use in qNMR [26]. In cases where the addition of an additional components can affect the sample, the referent compound can be positioned externally or an artificial referent signal can be generated by an NMR instrument such as ERETIC (electronic referencing to access in vivo concentrations), PIG (pulse into gradient), ARTSI (amplitude-corrected referencing through signal injection), PULCON (pulse length-based concentration measurements) and QUANTAS (quantification by artificial signal) [27].

The reliability and accuracy of concentration determination using qNMR depend on the experiment parameters setup and consideration of sample conditions, which is extensively covered elsewhere [28,29]. In reality, the experimental setup can be optimized to reduce the quantitative inaccuracy of qNMR below 2.0 %, an acceptable limit for precise and accurate quantification[30]. Furthermore, the NMR concentration determination can be achieved from low micromolar concentrations 4 μM even if the solvent water peak is used as the concentration reference [31]. This approach expands the applicability domain of the qNMR as the effects of deuterated water on p*K*a, log *P* and other physico-chemical parameters are removed.

### Drug solubility

**Figure 2.**
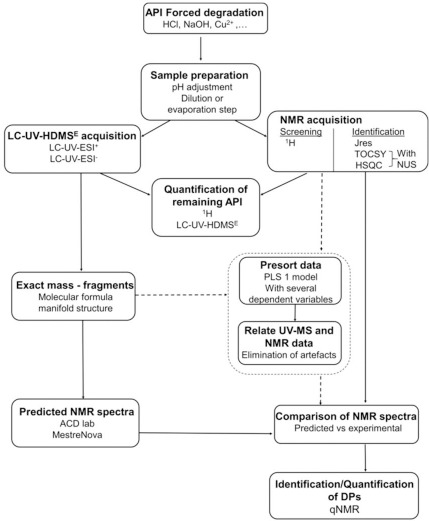
Workflow of the mass spectrometric-nuclear magnetic resonance (MS-NMR) approach. Taken from [33]. Copyright (2019) from MDPI under Creative Commons Attribution (CC BY) license.

Drug solubility is one of the key properties that influences the permeability and concentration of the active pharmaceutical ingredient in the physiological conditions after administration. While the HPLC measurements are generally used to determine solubility of drugs, as one of the most reliable and sensitive methods, it may be relatively slow process as often requires method development and creation of a calibration curve for quantitation. The need for faster measurements of large number of compounds, often with limited amounts, qNMR can be used to develop faster methods for solubility measurement, even in complex matrices that may affect drug solubility. As the NMR is fast method with relatively simple sample preparation procedure, it can be used to determine drug solubility in simple formulations, by calculation the integration ratio of the drug signal to the internal water soluble standard 3-(trimethylsilyl)propionic-2,2,3,3-d4 acid, sodium salt signal [32]. The further advantage is that the concentrations of all hydrogen containing components of the formulation could be determined simultaneously, along with detecting possible impurities. The new peaks that appear in samples, possibly during the degradation studies, can be quantified by NMR and further characterized by a combination of NMR spectroscopy and mass spectrometry (MS) protocol (Figure 2) [33]. Additionally, qNMR also allows determination of drug concentrations in presence of excipients without separation and at different temperatures [34].

Often, quantification of the concentration of an active pharmaceutical ingredient in complex mixtures is impeded in one-dimensional hydrogen (1D ^1^H) NMR spectra due to the signal overlap. The addition in signal separation along the second dimension in two-dimensional (2D) NMR spectra generally leads to reduction of signal overlap. The 2D COrrelation SpectroscopY (COSY) NMR spectra can be used for quantification purposes by integrating the volumes of crosspeaks of metabolites, that are proportional to their concentrations [35]. There is a plethora of different homo- and heteronuclear experiments that can be used to obtain quantitative information about sample components. Although this relationship between the peak volume and the concentration is not as simple as in 1D NMR spectroscopy, the volume of the signal depends on various peak relevant parameters, including relaxation times, coupling between different nuclei and pulse sequence parameters, there is a range of strategies that can be employed to achieve absolute quantification [36,37]. However, the need for integration of the peak volumes and careful validation of 2D methods employed[38], are limiting factors that may affect applicability of 2D qNMR spectroscopy as a fast and simple method. Alternatively, diffusion-ordered NMR spectroscopy (DOSY) can be used as 2D method that detects not only association between different components that may include small compounds and polymers [39], but it can be used for quantification too. Diffusion coefficients, determined by DOSY, can be used to evaluate solubilisation capacity of micelles of sodium dodecyl sulphate (SDS) for poorly soluble molecules such as artemisinin and curcumin. The solubility of these molecules can be evaluated, individually and in combination of different components by plotting selected chemical shifts against the diffusion coefficients determined by 2D DOSY spectra [40]. Furthermore, beside the information on solubility of investigated compounds in fasted state simulated intestinal fluid, the DOSY spectra provide information on diffusion properties of micelles formed by bile acids and poorly soluble drugs [41].

### Log P determination

The partition coefficient (*P*) reflects the different solubility of a molecule in two immiscible phases. The logarithm of P (log *P*) determined for water and octanol is often correlated with lipophilicity and of the molecules and indirectly with its permeability through biological barriers. The log *P* determination requires measuring equilibration concentrations of the molecule in water and octanol. The above-mentioned advantages of NMR, direct detection of analyte without reliance on presence of a chromophore, simple data interpretation, and minimal sample preparation, can be used in determination of log *P*. If a drug, or a drug candidate, has the aqueous solubility higher than the limit of quantification by qNMR, its concentration in water and octanol can be determined directly after separation and without the need for calibration curve [42]. This approach can be applied after compound partitioning between water and 1-octanol using standard methods by taking aliquots from two layers. The signal amplitudes extracted the a compound from each can be analyzed using the CRAFT software, that directly calculates both log *P* and log *D*_7.4_ values [43].

**Figure 3.**
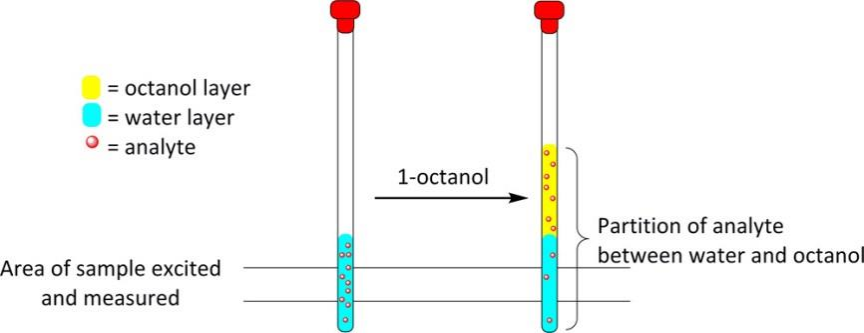
Schematic diagram of the analyte partition between the water and 1-octanol layers upon equilibration. Taken with permission from [44] (https://doi.org/10.1021/acsomega.7b01102) and further requests for permission should be directed to ACS. .

The similar experiment can be conducted directly in the NMR tube by mini shake-flask approach, where the initial determination of equilibrium concentrations of an analyte in water is followed by addition of the octanol layer and equilibration of the sample (Figure 3). The measurement the concentrations of the analyte A in the water phase before and after equilibration. The concentration of the analyte can be calculated as the difference between the amount of analyte in water before and after equilibration step [44]. This approach could easily be implemented as a follow up step to solubility determination of drug candidates and be possibly incorporated in automation assays that use NMR spectroscopy.

Measurement of log *P* for fluorinated compounds can employ ^19^F NMR spectroscopy in addition to ^1^H NMR spectroscopy. Using similar procedures to previously described procedures, there is no need for calibration curves providing a straightforward method for the measurement of lipophilicity of fluorinated compounds [45,46].

### pKa determination

**Figure 4.**
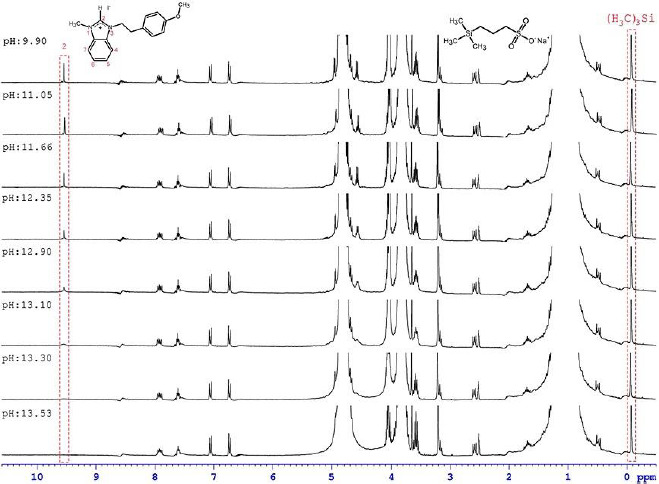
Different pH value ^1^H NMR spectrums obtained by the 10 mM tetrabutylammonium hydroxide solution (TBAH) titration of 1-[2-(4-methoxyphenyl)ethyl]-3-methylbenzimidazolium iodide (2) compound in 10 mM solution and the sigmoidal curve that was acquired with the passing of integral values obtained from these spectrums to the against pH values. Taken with permission from [48]. Copyright (2019) with permission from Wiley.

The acid-base dissociation constant (p*K*a) of a drug is also a physicochemical parameter that affects many of its biophysical characteristics., including solubility, lipophilicity, binding to proteins and crossing biological barriers. The p*K*a determination involves monitoring the ionization state of a molecule with changing pH. Traditional methods, such as potentiometric titrations or following the change of UV absorption as a function of pH, involve complex experimental setup and analyses of results. NMR spectroscopy is also based on analysis of protonation degree of a molecule with changing pH. The protonation degree is monitored either by the chemical shift changes close to the acidic or basic regions of molecule [47] or via changes of the integration values of peaks that arise from ionizable groups (Figure 4) [48].

## Conclusions

The NMR spectroscopy is extensively used in the various drug discovery and development process, however further applications of qNMR can have a significant impact on the early stages of selection of lead compounds with adequate ADME properties. Furthermore, the determination of physico-chemical properties is not affected by the presence of other components, moreover it may provide significant information on behaviour of molecules in environments that are more similar to conditions in *in vitro* and possibly *in vivo* experiments. Despite first NMR signals discovered over 70 years ago, the development of new approaches in solving of real-life problems is still undergoing and it has a potential to play even greater role in the future drug discovery programmes.
